# An Improved Genetic Algorithm for Location Allocation Problem with Grey Theory in Public Health Emergencies

**DOI:** 10.3390/ijerph19159752

**Published:** 2022-08-08

**Authors:** Shaoren Wang, Yenchun Jim Wu, Ruiting Li

**Affiliations:** 1 Business School, Huaqiao University, Quanzhou 362021, China; 2 MBA Program in Southeast Asia, National Taipei University of Education, Taipei City 10671, Taiwan; 3 Graduate Institute of Global Business and Strategy, National Taiwan Normal University, Taipei City 10645, Taiwan

**Keywords:** emergency medical facility location-allocation problem (EMFLAP), genetic algorithm, public health emergency, emergency logistics, grey theory

## Abstract

The demand for emergency medical facilities (EMFs) has witnessed an explosive growth recently due to the COVID-19 pandemic and the rapid spread of the virus. To expedite the location of EMFs and the allocation of patients to these facilities at times of disaster, a location-allocation problem (LAP) model that can help EMFs cope with major public health emergencies was proposed in this study. Given the influence of the number of COVID-19-infected persons on the demand for EMFs, a grey forecasting model was also utilized to predict the accumulative COVID-19 cases during the pandemic and to calculate the demand for EMFs. A serial-number-coded genetic algorithm (SNCGA) was proposed, and dynamic variation was used to accelerate the convergence. This algorithm was programmed using MATLAB, and the emergency medical facility LAP (EMFLAP) model was solved using the simple (standard) genetic algorithm (SGA) and SNCGA. Results show that the EMFLAP plan based on SNCGA consumes 8.34% less time than that based on SGA, and the calculation time of SNCGA is 20.25% shorter than that of SGA. Therefore, SNCGA is proven convenient for processing the model constraint conditions, for naturally describing the available solutions to a problem, for improving the complexity of algorithms, and for reducing the total time consumed by EMFLAP plans. The proposed method can guide emergency management personnel in designing an EMFLAP decision scheme.

## 1. Introduction

Public health emergencies have increased in frequency on a global scale over the past decades. Some of the most renowned emergencies include the SARS outbreak in China in 2003 and the H1N1 flu outbreak that started in the United States in 2009 and spread across 214 countries and regions. In 2013, the H7N9 epidemic caused panic among the public [1]. The occurrence of these public health emergencies is usually followed by a shortage of specialized hospitals and hospital beds. A recent example of a major public health emergency is the COVID-19 outbreak at the end of 2019, which led to an extreme shortage of emergency medical facilities and imposed a major challenge to the global public health emergency system due to its fast propagation, broad scope of infection, and difficult prevention and control. Therefore, the location-allocation problem (LAP) of emergency facilities has become an important research problem in emergency management. An effective mode of medical rescue after the outbreak of a major public health emergency relies on the timely transportation of medical supplies and workers to the field using modern equipment, such as intelligently extended vehicle-mounted mobile cabin hospitals or folding portable cabin hospitals. Following the COVID-19 outbreak, the Chinese government expedited the construction of over 20 mobile cabin hospitals in Wuhan to control the further spread of the virus. As the virus spread across the globe, other countries, such as Russia, Korea, Italy, Iran, and Germany, started building their own emergency medical facilities. Mobile cabin hospitals have proven very useful in the quarantine, providing facilities for accommodation, testing, and medical treatment of confirmed and suspected COVID-19 cases. Responding to this public health emergency in time can aid in the rapid recovery of infected persons, thus greatly reducing pandemic-related deaths and curbing the spread of the virus. In this case, the making of emergency medical facility location-allocation decisions has received much attention from academia, the government, and society at large.

Introduced by Cooper [2] in 1963, the concept of LAP has aroused several researches in the context of public health emergencies. Recent theoretical studies on LAP in logistics systems have taken several constraint conditions into account, including waiting time, facility capacity limitations, and service time. Previous studies have further extended LAP into random LAP and multistage LAP and have mainly focused on the various constraint conditions of business logistics systems and the improvement of solving algorithms. However, only few researchers have paid attention to the complexity of public health emergency logistics and the LAP of emergency medical facilities in special scenarios. To fill this gap, this study considers the influence of the number of infected persons in pandemic-stricken areas on the demand for emergency medical facilities. To minimize the total emergency rescue time on the precondition that all patients are treated, the LAP of emergency medical facilities was selected as the research object, an LAP optimization model was built, and the corresponding algorithm was proposed to formulate an emergency medical facility location-allocation optimization scheme for emergency management departments.

As mentioned, the research problem in this study is a generalization of emergency medical facility location-allocation problem (EMFLAP) model, which is an extension of the facility location-allocation problem (FLAP). The objective function of the model is to minimize the total time of relief operations for EMFLAP plans, and the problem involves a location decision for optimally locating emergency medical facilities and an allocation decision to allocate the affected area’s patients to emergency medical facilities, i.e., which emergency medical facilities to open and how to assign the patients to the opened emergency medical facilities. Due to the cases uncertainty in public health emergencies, how to predict the number of affected persons in a disaster-struck area becomes necessary. Therefore, accounting for the uncertainties of model parameters for EMFLAP, the technique of the grey forecasting model is introduced to deal with the issue of forecasting cases associated with each affected area. These problems are one of the most critical strategic decisions for emergency management personnel.

This study offers several contributions to the literature. First, an appropriate forecasting method was adopted to calculate the level of demand for emergency facilities, and an LAP optimization model of emergency medical facilities in the context of public health emergencies was constructed. Second, a serial-number-coded genetic algorithm (SNCGA) was proposed, which not only is capable of naturally solving certain problems and processing complex model constraint conditions but is also convenient for the dynamic variation operation of genetic algorithms (GAs). This process simplifies the calculation of the objective function, reduces the calculation complexity, and verifies the feasibility and validity of algorithms.

The rest of this study is organized as follows. Section 2 reviews the relevant literature and distinguishes this study from previous works. Section 3 predictively analyzes the development trend of major public health emergencies in the context of pandemics, builds an LAP model of emergency medical facilities, designs an improved GA according to the model features, and compares the coding performance of this algorithm with that of a simple (standard) genetic algorithm (SGA). Section 4 improves the SGA, uses the improved GA to solve the emergency medical facility LAP model in Wuhan, China, as a calculation example, and compares these two algorithms in terms of their calculation time and objective function results. Section 5 concludes the study and proposes directions for future research.

## 2. State of the Art

Numerous theoretical studies on LAP optimization have been reported. The theoretical discussions on the various types of LAP optimization models and their improved algorithms in business logistics systems have also expanded to include the topic of LAP optimization in emergency logistics systems.

Following the introduction of facility LAP, LAP optimization models and algorithms, mainly including multistage LAP, multi-objective LAP, and random LAP, have been widely studied in business logistics systems research. George et al. [3] examined the multistage facility LAP and proposed a mixed-integer programming approach and a dynamic programming approach. Ghodratnama et al. [4] developed a bi-objective new hub location-allocation model for congestion and production scheduling. Hajipour et al. [5] proposed a multi-objective multi-layer facility location-allocation model with congested facilities using classical queuing systems, a Pareto-based multi-objective meta heuristic approach based on the multi-objective vibration damping optimization, and the multi-objective harmony search algorithm to find and analyze Pareto optimal solutions. Constantine et al. [6] proposed the emergency facility location problem, which has been extensively studied in succeeding research. Khalili-Damghani et al. [7] proposed a bi-level two-echelon mathematical model to minimize pre-disaster costs and maximize post-disaster relief coverage areas and to determine the optimal number and location of distribution centers while minimizing the inventory costs of relief supplies. Specific to the emergency logistics system of the U.S. Federal Emergency Management Agency, Afshar et al. [8] proposed a mathematical model that controls the flow of several relief commodities from different sources through the supply chain and until they were delivered to the recipients. Considering the psychological punishment cost of victims, Hu et al. [9] developed novel mathematical programs for multi-step evacuation and sheltering with the goal of minimizing monetary costs and psychological penalties.

The above studies have built emergency facility location decision models from different angles and designed the corresponding rescue schemes. Multi-objective optimization models are often used by researchers to improve the efficiency and effectiveness of humanitarian rescue facilities, including refuges, medical centers, warehouses, and distribution centers. Baharmand et al. [10] proposed a bi-objective multi-layer location-allocation model that could help temporary distribution centers make location-allocation decisions when responding to sudden disasters. Abounacer et al. [11] solved a three-objective location-transportation problem by determining the number, the position, and the mission of required humanitarian aid distribution centers (HADC) within the disaster-struck region and by dealing with the distribution of aid from HADCs to demand points. They also proposed an epsilon-constraint method for addressing this problem and proved that this solution could generate the exact Pareto front. Barzinpour et al. [12] developed a new multi-objective mixed-integer linear programming model to solve the emergency facility location problem and the allocation of affected people to local emergency management facilities. Loree et al. [13] developed a mathematical model that determined the location of distribution points and the allocation of inventories in post-disaster humanitarian logistics. Praneetpholkrang et al. [14] proposed a multi-objective optimization model for determining the allocation of shelters in response to humanitarian relief logistics and then used the epsilon constraint method and goal programming (GP) to solve this model. Considering factors such as the level of decision making, the utilization efficiency, and capacity constraints of shelters, He et al. [15] constructed a bi-level multi-objective location-allocation model. Ghasemi et al. [16] proposed an uncertain multi-objective multi-commodity multi-period multi-vehicle location-allocation mixed-integer mathematical programing model for the response phase of earthquakes and then solved this model by using the modified multiple-objective particle swarm optimization (PSO), non-dominated sorting GA-II, and epsilon constraint method. They found that the multi-objective PSO algorithm was superior to the other solving methods.

An emergency medical facility location-allocation scheme is urgently needed following the declaration of a public health emergency. LAP is an NP-hard problem [17,18] that can be effectively solved by heuristic algorithms. The proposed effectiveness means that solutions can be obtained using the heuristic algorithm within a reasonable computational time. The simplex algorithm, widely used weighted-sum (WS) approach, and ∈-constraint (EC) method work well for small-sized instances, but they have difficulty in solving large-sized instances due to the NP-hardness of the studied problem [19]. Thus, several heuristic algorithms (e.g., ant colony optimization, genetic algorithm, and particle swarm optimization, etc.) have been proposed to solve location allocation problems, each of which has several limitations, such as accuracy, time, and flexibility, besides their advantages. Thus, a large amount of solution approaches for different models have been proposed in the past decades. A series of heuristic algorithms, including hybrid algorithms, have also been developed to solve complicated LAP. For instance, a hybrid method of genetic algorithm and sub-gradient technique is used to solve the problem efficiently [20]; a hybrid intelligent algorithm that integrates the simplex algorithm, fuzzy simulations, and a genetic algorithm is produced [21]; and an ant colony optimization (ACO)-based heuristic is developed for solving the model [22]. Sharma et al. [23] developed a tool called hybridization or integration of different techniques to locate temporary blood banks during and after a disaster that could serve hospitals in the shortest possible response time and then used the tabu search heuristic method to calculate the optimal number of temporary blood centers while considering the cost components. Given the disaster-induced demands and the available transportation infrastructure in disaster response, Chen et al. [24] applied integer programming and network-based partitioning to determine temporary locations for on-post emergency medical service facilities and adapted Lagrangian relaxation to extend this problem further to a larger scale. To respond quickly to emergencies, Toro-Díaz et al. [25] developed a mathematical formulation that combines an integer programming model representing location and dispatching decisions with a hypercube model representing the queuing elements and congestion phenomena and then proposed an optimization framework based on GA. Therefore, using heuristic (intelligent) algorithms (e.g., ant colony optimization, genetic algorithm, and particle swarm optimization, etc.) can effectively solve the problems. To this end, a serial-number-coded genetic algorithm (SNCGA) is designed for solving the problem in the study.

Researchers have solved all kinds of facility LAPs by using heuristic algorithms, such as the tabu search heuristic method, PSO, and GA, etc. However, the calculation efficiency of these algorithms needs improvement. For instance, the standard GA, also known as SGA, has a very long chromosome string length due to the adopted coding scheme. This approach also involves complex calculations and consumes large memory, which are not conducive to reducing the algorithm complexity, processing constraint conditions, executing genetic operations, and reducing the algorithm calculation time. Some researchers have improved GA [26,27], but the early-maturing problem of GA has also been addressed inefficiently in previous research.

Major public health emergencies are known for their suddenness, complexity, and destructiveness, all of which introduce huge challenges in rapid emergency response. Emergency medical facility security work serves as the foundation for defeating pandemics, and the basis for this security work lies in an accurate demand forecasting. The demand for emergency medical facilities is positively correlated with the number of infected persons and can be estimated by predicting the number of infected persons after the occurrence of a major public health emergency. The number of infected persons has been forecasted by some researchers using big data and various forecasting models, which provide a reference for making decisions in rescue work. Ginsberg et al. [28] analyzed large numbers of Google search queries to track influenza-like illnesses in a population and accurately estimate the current level of weekly influenza activity in each region of the U.S. Wu et al. [29] used a susceptible-exposed-infectious-recovered (SEIR) metapopulation model to predict the cross-infection probability of infectious diseases and used Markov chain Monte Carlo methods to estimate the basic reproductive number. On the basis of the relationship between the spread of viruses and social networks, Nikakhtar et al. [30] developed a simulation model wherein a virus spreads across a social network, and the infected patients were directed to a healthcare system. However, the validity and reliability of big data (e.g., Google search)-based flu forecasting cannot be easily verified (Lazer et al.) [31]. Therefore, the evolution trends of public health emergencies have been forecasted by some researchers by using corrected infectious disease transmission dynamics SEIR models. To estimate parameters from the time series data on daily incidence and mortality associated with the Ebola outbreak in the Democratic Republic of Congo in 1995, Lekone et al. [32] developed a stochastic discrete-time SEIR model for infectious diseases. Boldog et al. [33] developed a computational tool to assess the risk of COVID-19 outbreaks outside China and then estimated the dependence of the risk of a major outbreak in a country from imported cases on key parameters. To estimate the COVID-19 epidemiology curve, Andelic et al. [34] utilized the artificial intelligence (AI) method called genetic programming (GP) algorithm to develop a symbolic expression (mathematical equation) that can be used for the estimation of the epidemiology curve for the entire U.S. with high accuracy. While these researchers have used specific models or algorithms to predict the occurrence and development of infectious diseases and to analyze epidemic trends, the timeliness and accuracy of their forecasting methods hardly meet the demand of major public health emergencies.

The above literature review reveals that: (1) the existing studies have built emergency facility location decision models from different angles and designed the corresponding rescue schemes; however, emergency facility location-allocation decisions have been rarely studied; (2) the number of infected persons in public health emergencies has been independently studied, or the emergency facility LAP has been independently explored in previous research; and (3) researchers have solved all kinds of facility LAPs by using heuristic algorithms; however, the calculation efficiency of these algorithms needs improvement. Leknes et al. [35] presented a new mixed-integer model for this problem that is particularly suitable for regions with heterogeneous demand and multiple performance measures. This model decides the locations/allocation of stations/ambulances and then calculates the service and arrival rates for each station and the probabilities that a call is served by a particular station. However, emergency facility location-allocation decisions have been rarely studied based on emergency facility demand forecasting. Therefore, an appropriate forecasting method was used in this study to predict the number of infected persons and to further calculate the demand for of emergency medical facilities. An emergency medical facility location-allocation optimization model and algorithm were also proposed to improve emergency rescue efficiency.

## 3. Methodology

### 3.1. Problem Description and Assumptions

#### 3.1.1. Problem Description

**Figure 1:**
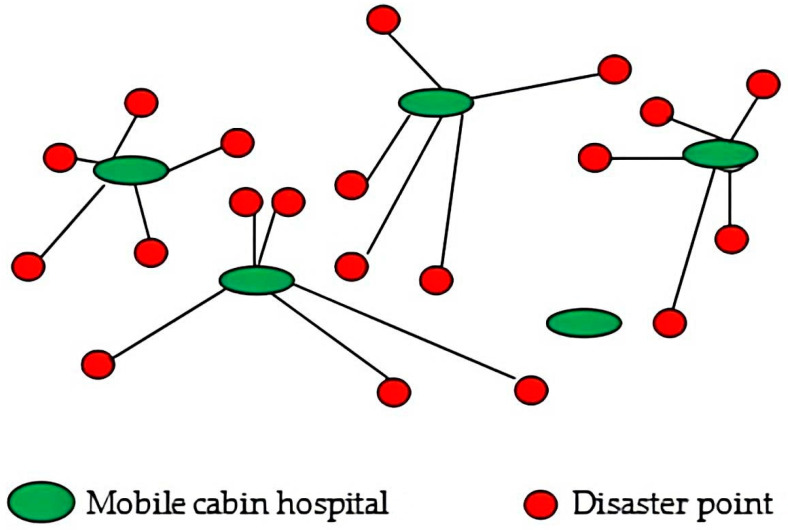
Location-allocation problem diagram.

In response to major public health emergencies, temporary emergency medical facilities, such as mobile cabin hospitals, should be built to treat the patients, prevent the spread of diseases, and reduce the associated mortality. An important link in rescue operations during major public health emergency is the selection of an appropriate method for predicting the number of affected persons in disaster-struck areas and for making informed decisions that can guide the design of an emergency medical facility location-allocation scheme. This link directly affects the prevention and control of pandemics. Figure 1 illustrates the location-allocation network of mobile cabin hospitals.

#### 3.1.2. Assumptions

On the basis of the aforementioned features, the following assumptions were made: (1) each infected patient needs a hospital bed. Therefore, the total number of emergency medical facilities (i.e., mobile cabin hospitals) can be inferred by predicting the number of cases; and (2) according to the current situation, the construction period of newly built and rebuilt mobile cabin hospitals is already known. The definitions of symbols used throughout this study are presented below:

Parameters:

- C={m|m=1,2,3,…,M} is a set of disaster points;
- W={p|p=1,2,3,…,P} is a set of potential mobile cabin hospitals;
- $WT_{p}$ denotes the time spent in constructing the temporary mobile cabin hospital *p*;
- $WQ_{p}$ represents the maximum admission capacity of potential mobile cabin hospital *p*;
- $d_{mp}$ denotes the distance between the affected area m and the potential mobile cabin hospital *p*, $d_{mp}=\sqrt{{\left(x_{m}-x_{p}\right)}^{2}+{\left(y_{m}-y_{p}\right)}^{2}}$, where $x_{m}$ and $y_{m}$ represent the *x* and *y* coordinates of node *m*, respectively;
- t is the average time spent in transporting each infected patient from a disaster point to a mobile cabin hospital per unit and distance;
- $\vartheta_{m}$ denotes the population at disaster point *m*;
- $q_{m}$ is the number of infected persons at disaster point *m*, $q_{m}=\rho \vartheta_{m}$ where ρ is the infection rate calculated by dividing the accumulative number of infected persons at a time point by the total population.

Decision variables:

- ${\hat{x}}^{\left(0\right)}$ denotes the restored values of the grey forecasting model;
- $y_{p}$ equals 1 if the potential mobile cabin hospital *p* is selected and equals 0 otherwise;
- $\alpha_{mp}$ equals 1 if the affected area *m* is assigned to the selected mobile cabin hospital *p* and equals 0 otherwise.

### 3.2. Grey Model and Tests

The basic principles and modelling mechanism of GM (1, 1) is discussed as follows [36,37]:

Step 1. For *n* (*n* ≥ 4) samples, the original time sequence, $X^{\left(0\right)}$, is given as: $X^{\left(0\right)}=[x^{\left(0\right)}(1),x^{\left(0\right)}(2),x^{\left(0\right)}(3),\ldots x^{\left(0\right)}(n)]$, then $X^{\left(1\right)}=[x^{\left(1\right)}(1),x^{\left(1\right)}(2),x^{\left(1\right)}(3),\ldots x^{\left(1\right)}(n)]$ is called the accumulating generation operator (AGO) sequence of $X^{\left(0\right)}$, where $x^{\left(1\right)}(k)=\sum_{i=1}^{k}x^{\left(0\right)}(i),k=1,2,3,\ldots ,n$, and $z^{\left(1\right)}(k)=0.5\times (x^{\left(1\right)}(k)+x^{\left(1\right)}(k-1)),k=2,3,\ldots ,n$ is called the mean generation of consecutive neighbors sequence of $X^{\left(1\right)}$.

Step 2. The first-order grey differential equation can be constructed as follows:

(1) $$x^{\left(0\right)}(k)+az^{\left(1\right)}(k)=b,$$

where *a* and *b* are the development and control coefficients, respectively. These coefficients can be obtained as follows using the least-squares estimation method: $\hat{\alpha }={\left(a,b\right)}^{T}={\left(B^{T}B\right)}^{-1}B^{T}Y$, where $\hat{\alpha }$ is a sequence of parameters, $B=\left[\begin{array}{l} -Z^{\left(1\right)}(2),1\\ -Z^{\left(1\right)}(3),1\\ \ldots \\ -Z^{\left(1\right)}(n),1 \end{array}\right]$ and $Y=\left[\begin{array}{l} x^{\left(0\right)}(2)\\ x^{\left(0\right)}(3)\\ \ldots \\ x^{\left(0\right)}(n) \end{array}\right]$. The mean sequence generating equation of Equation (1) is called the differential equation, which can be derived as

(2) $$\frac{dx^{\left(1\right)}}{dt}+ax^{\left(1\right)}=b,$$

Step 3. Following the above equation, the grey prediction equation can be described as follows:

(3) $${\hat{x}}^{\left(1\right)}(k)=[x^{\left(0\right)}(1)-\frac{b}{a}]e^{-a(k-1)}+\frac{b}{a}$$

where ${\hat{x}}^{\left(1\right)}(k)$ indicates the prediction of $x^{\left(1\right)}(k)$ at time point k and the initial condition, ${\hat{x}}^{\left(0\right)}(1)=x^{\left(0\right)}(1)$. The inverse AGO (IAGO) sequence can be obtained as follows:

(4) $${\hat{x}}^{\left(0\right)}(k)={\hat{x}}^{\left(1\right)}(k)-{\hat{x}}^{\left(1\right)}(k-1)=(1-e^{a})\left[x^{\left(0\right)}(1)-\frac{b}{a}\right]e^{-a(k-1)},k=2,3,\ldots ,n,$$

Although the grey forecasting model can effectively solve the system forecasting problem induced by small amount of information, this model cannot easily predict sequences because the number of infected cases is influenced by all kinds of factors after the occurrence of a public health emergency. Therefore, the following tests should be performed:

- Stepwise ratio test: To test the appropriate regularity of a data sequence, let $L(k)=x^{\left(0\right)}(k-1)/x^{\left(0\right)}(k),k=2,3,\ldots ,n$. More L(k) values falling between $e^{-\frac{2}{n-1}}$ and $e^{\frac{2}{n+1}}$ indicates a better modeling effect; that is, when $e^{-\frac{2}{n-1}}\leq L(k)\leq e^{\frac{2}{n+1}}$, the GM (1, 1) modeling effect is the best;
- Accuracy test: Let $\varepsilon^{\left(0\right)}=\left\{\varepsilon^{\left(0\right)}(1),\varepsilon^{\left(0\right)}(2),\ldots ,\varepsilon^{\left(0\right)}(n)\right\}$ be the absolute error, where $\varepsilon^{\left(0\right)}(k)=x^{\left(0\right)}(k)-{\hat{x}}^{\left(0\right)}(k),k=1,2,\ldots ,n$. Then, the relative error, mean relative error, mean absolute error, and variance are $\Delta_{k}=\left|\frac{\varepsilon^{\left(0\right)}(k)}{x^{\left(0\right)}(k)}\right|$, $\bar{\Delta }=\frac{1}{n}\sum_{k=1}^{n}\Delta_{k}$, $\bar{\varepsilon }=\frac{1}{n}\sum_{k=1}^{n}\varepsilon^{\left(0\right)}(k)$, and $\delta^{2}=\frac{1}{n-1}\sum_{k=1}^{n}{\left(\varepsilon^{\left(0\right)}(k)-\bar{\varepsilon }\right)}^{2}$, respectively. Let $\bar{x}=\frac{1}{n}\sum_{k=1}^{n}x^{\left(0\right)}(k)$ and $s_{1}^{2}=\frac{1}{n-1}\sum_{k=1}^{n}(x^{\left(0\right)}(k)-\bar{x}{)}^{2}$ be the mean value and variance of the original data, respectively. Then, the ratio of the mean square error and small error probability are μ=δs1 and $\varphi \left(\left|\varepsilon^{\left(0\right)}(k)-\bar{\varepsilon }\right|<0.6745s_{1}\right)$, respectively. To test the similarity and proximity of the simulated and original data, let $\left|s\right|=\sum_{k=2}^{n-1}\left|x^{\left(0\right)}(k)\right|+\frac{1}{2}\left|x^{\left(0\right)}(n)\right|$, $\left|\beta \right|=\sum_{k=2}^{n-1}\left|{\hat{x}}^{\left(0\right)}(k)\right|+\frac{1}{2}\left|{\hat{x}}^{\left(0\right)}(n)\right|$, and $\left|\beta -s\right|=\sum_{k=2}^{n-1}\left|{\hat{x}}^{\left(0\right)}(k)-x^{\left(0\right)}(k)\right|+\frac{1}{2}\left|{\hat{x}}^{\left(0\right)}(n)-x^{\left(0\right)}(n)\right|$. The relevancy degree is derived as $\gamma_{s\beta }=\frac{1+\left|s\right|+\left|\beta \right|}{1+\left|s\right|+\left|\beta \right|+\left|\beta -s\right|}$. The model equation shows good forecasting accuracy and can be used in predicting future data when the mean relative error $\bar{\Delta }$ ≤ 0.01, the ratio of mean square error μ ≤ 0.35, the small error probability φ ≥ 0.95, and the relevancy degree $\gamma_{s\beta }$ ≥ 0.90.

### 3.3. EMFLAP Model and Solution

According to the above definitions, the total number of emergency medical facilities (i.e., mobile cabin hospitals) was inferred via epidemic forecasting through the grey forecasting model (Equation (4)). Emergency medical facility LAP (EMFLAP) optimization models were then constructed as follows:

(5) $$\min\;Z=\sum_{p\in W}WT_{p}\times y_{p}+\sum_{p\in W}\sum_{m\in C}t\times q_{m}\times d_{pm}\times \alpha_{mp}$$

(6) $$s.t.{}^{}\sum_{m\in C}q_{m}\alpha_{mp}\leq WQ_{p},\forall p\in W$$

(7) $$\sum_{m\in C}\alpha_{mp}\geq y_{p},\forall p\in W$$

(8) $$\alpha_{mp}\leq y_{p},\forall m\in C,p\in W$$

(9) $$\sum_{p\in W}\alpha_{mp}=1,\forall m\in C$$

(10) $$\sum_{p\in W}y_{p}\leq P,\forall p\in W$$

(11) $$\sum_{p\in W}WQ_{p}y_{p}\geq {\hat{x}}^{\left(0\right)}(k)-{\hat{x}}^{\left(0\right)}(k-1),\forall k=2,3,\ldots ,n$$

(12) $$y_{p}\in \left\{0,1\right\},\alpha_{mp}\in \left\{0,1\right\},\forall p\in W,m\in C$$

where objective function (5) indicates the minimum time consumed by an emergency medical facility location-allocation scheme in case of a public health emergency, including the time to construct mobile cabin hospitals and the time spent in transporting the infected patients. Equation (6) indicates that the total number of infected patients allocated from all disaster points to a mobile cabin hospital does not exceed the maximum number of patients that can be accommodated by this hospital. Equations (7) and (8) suggest that once established, each mobile cabin hospital needs to serve disaster points that can only be allocated to already-built mobile cabin hospitals. Equation (9) shows that each disaster point is served and only allocated to one mobile cabin hospital. Equation (10) shows that the number of established mobile cabin hospitals does not exceed the total number of potential hospitals. Equation (11) reveals that the total capacity of built mobile cabin hospitals should meet the demand of newly infected patients. Equation (12) represents a decision variable constraint.

LAP is an NP-hard problem that cannot be easily solved using an accurate algorithm in case this problem has a large scale. However, this problem can be effectively solved using intelligent algorithms. On the basis of the above model features, this study designed SNCGA, which has several obvious advantages over the GAs used by Mokhtarzadeh et al. [18] and Tang et al. [19] to solve LAP. The specific steps in algorithm are discussed as follows:

Step 1. Setting of algorithm parameters: Let Po, GP, Maxgen, Pc, and Pm be the number of individuals in the population (population size), generation gap, maximum number of generations, crossover rate, and mutation rate, respectively.

**Figure 2:**
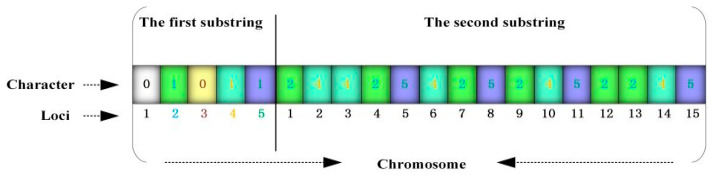
Schematic diagram of the coding technology in SGA.

Step 2. Chromosome coding and construction of initial population: SGA is a population-type operating algorithm that is mostly coded using a binary-coded chromosome, where binary symbols 0 and 1 indicate that the facility is not established and is established, respectively. The EMFLAP with M rescue points and P potential emergency medical facilities is coded as $\left\{x_{1},x_{2},x_{3},\ldots ,x_{P},{x^{\prime }}_{1},{x^{\prime }}_{2},{x^{\prime }}_{3},\ldots ,{x^{\prime }}_{M}\right\}$. In this way, the length of every chromosome string is P + M digits and comprises two substrings, where the genic value of the first substring is either 0 or 1 (indicating that the facility is not established or is established) and that of the second substring is the corresponding genic loci when the genic value of the first substring is 1 (indicating the allocation of established emergency medical facilities to the disaster points). The decision parameters are coded accordingly. The coding results under P = 5 and M = 15 are displayed in Figure 2. The 2nd, 4th, and 5th emergency medical facilities in the substring 1 are then built; the established emergency medical facility 2 in substring 2 is allocated to the 1st, 4th, 7th, 9th, 12th, and 13th disaster points; the established facility 4 is allocated to the 2nd, 3rd, 6th, 10th, and 14th disaster points; and the established facility 5 is allocated to the 5th, 8th, 11th, and 15th disaster points. Although binary coding, which is commonly used by GA, can indicate whether an emergency facility is established, this approach cannot reflect the structural characteristics of the problem to be solved. Therefore, the problem of allocating emergency facilities to disaster points needs to be addressed. The GA search space will sharply increase when this problem has a large scale.

A Po-row and P-column matrix Chrom1 with elements of 0 and 1 is constructed, and a Po-row and M-column random matrix Chrom2 with elements of 1-P is established using the randint function. Chrom = [Chrom1, Chrom2] is then defined as the established initial population.

**Figure 3:**
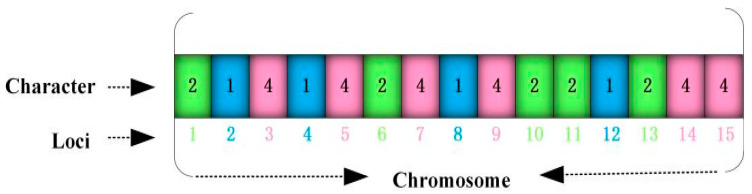
Schematic diagram of the coding technology in SNCGA.

For a specific application problem, how to design a perfect coding scheme is an important research direction of GA. To overcome the deficiency of SGA in binary coding, an improved GA algorithm called SNCGA was proposed to address the LAP of emergency medical facilities in the context of public health emergencies. The strength of SNCGA lies in coding natural numbers and combining them with the serial numbers at the corresponding locations. In other words, the natural number (genic value) of each chromosome (individual) may indicate whether an emergency medical facility has been established. The gene locus corresponding to this genic value is referred to as the serial number of a disaster point. Therefore, the correspondence between the genic value (serial number of an emergency medical facility) and gene locus (serial number of a disaster point) reflects the allocation of established emergency medical facilities to disaster points. Specifically, the EMFLAP with M disaster points and P potential emergency medical facilities can be coded as $\left\{x_{1},x_{2},x_{3},\ldots ,x_{M}\right\}$, and the length of each chromosome string comprises M digits. The coding results under P = 5 and M = 15 are presented in Figure 3. The mobile cabin hospital 1 is allocated to the 2nd, 4th, 8th, and 12th disaster points; the mobile cabin hospital 2 is allocated to the 1st, 6th, 10th, 11th, and 13th disaster points; and the mobile cabin hospital 4 is allocated to the 3rd, 5th, 7th, 9th, 14th, and 15th disaster points. Compared with the traditional GA, SNCGA offers the following advantages when solving LAP:

- SNCGA can conveniently handle the complex constraint conditions in the model. The union set of string characters represents a set of established mobile cabin hospitals (e.g., hospitals 1, 2, and 4 are established in Figure 3), and the capacity constraint of mobile cabin hospitals can be conveniently handled using the penalty function method. The string position reflects that all disaster points are allocated to cabin hospitals;
- Relative to the binary coding used by SGA to solve LAP, each chromosome string has P + M digits. The proposed serial-number-coding technology GA (SNCGA) only contains M digits, thereby greatly reducing the computation time and space and reducing the algorithm complexity. The efficiency of GA obviously increases along with the problem scale (quantities of P and M).

The Genetic Algorithm Toolbox for MATLAB, which was developed by the University of Sheffield, was used to generate an initial random population. First, the crtbase function was used to build a vector with a length of M and containing M basic characters (0, 1, 2,…, P − 1) with a base number of P. Second, a matrix (Chrom) with a random number of elements (the basic characters are decided by the corresponding vector) was created using the crtbp function plus 1 to ensure that the serial numbers of rows and columns are not equal to 0 during the matrix processing. The initial population (Chrom) was eventually obtained.

Step 3. Calculate fitness: Fitness f_i_ (Chrom_i_ (gen)) indicates the fitness of chromosome i of Chrom at the gen. This parameter is obtained by converting the objective function; that is, the fitness function f_i_ = 1/Z (i), Z (i) is the objective functional value of chromosome i.

Step 4. Genetic operations:

- Selection operation. Stochastic universal sampling was combined with a fitness-based reinsertion method (an elitist strategy);
- Crossover operation. A multi-points crossover operator (a crossover operator with great destruction was used) can help the algorithm search for the solution space. The disruptive nature of multi-point crossover appears to encourage the exploration of the search space rather than favoring the convergence to highly fit individuals early in the search, thus making the search more robust [38];
- Mutation operation. A real-valued mutation was used, and the range of mutation was restricted after adding the field descriptor to ensure that the boundaries of decision variables are not exceeded after the mutation.

Step 5. Termination conditions of the algorithm: When gen ≤ Maxgen, steps 3 and 4 were repeated. The algorithm terminates when gen > Maxgen.

## 4. Results Analysis

### 4.1. Data Acquisition

A map of Wuhan, China, was used as a planar graph to select the locations of disaster points and potential mobile cabin hospitals. Both the westernmost *x* coordinate and southernmost *y* coordinate of this map were equal to 0. On the basis of the maximum east-west transverse distance of 134 km and maximum south-north longitudinal distance of 155 km in Wuhan, the 0 km to 150 km range was selected as the 2D coordinate. The Huangpi, Huanghe, Leishenshan, Dongxihu, and Wuchang cabin hospitals were taken as standby newly built or rebuilt cabin hospitals denoted by A, B, C, D, and E, respectively. The central locations in 15 districts (Caidian, Hannan, Dongxihu, Hanyang, Qiaokou, Jianghan, Jiangan, Wuchang, Hongshan, Huangpi, Jiangxia, Qingshan, Xinzhou, East Lake Scenic Area, and East Lake High-Tech Development Zone) were chosen as disaster points numbered from M1 to M15. The parameters of these cabin hospitals and districts are listed in Table 1 and Table 2. The data of Wuhan from 1–5 February 2020 as provided by the National Health Commission were used as the basic data for forecasting (see Table 3 for more details). The permanent resident populations in the different districts of Wuhan are presented in Table 4 (all numbers are rounded-off; unit: ten thousand persons).

### 4.2. Case Study Results Analysis

Wuhan previously suffered from a great shortage of hospital beds during the onset of the COVID-19 outbreak. Many mild and moderate COVID-19 patients had to be sent home for quarantine and observation. However, the decision to construct the second batch of mobile cabin hospitals resulted in the wastage of medical resources. Therefore, forecasting the demand for emergency medical facilities is critical for the government to formulate scientific epidemic prevention and control decisions. Although the demand for emergency medical facilities cannot be easily predicted, demand forecasting remains the main basis for formulating epidemic prevention and control policies. According to the forecasting model GM (1, 1) in Section 3.2, the original time sequence $X^{\left(0\right)}$ = [4109, 5142, 6384, 8351, 10, 117] in Table 3 was used to obtain the concrete expression of the forecasted value sequence, namely Equation (4):

(13) $$\left\{ \begin{array}{l} {\hat{x}}^{\left(0\right)}(k)=x^{\left(0\right)}(1),k=1\\ {\hat{x}}^{\left(0\right)}(k)=(1-e^{-0.226})\left[x^{\left(0\right)}(1)-\frac{3657.823}{-0.226}\right]e^{0.226(k-1)},k=2,3,\ldots ,n \end{array}\right.$$

To verify the effect and accuracy of Equation (13), relevant tests were performed. The related index calculation results and criteria are listed in Table 5.

**Figure 4:**
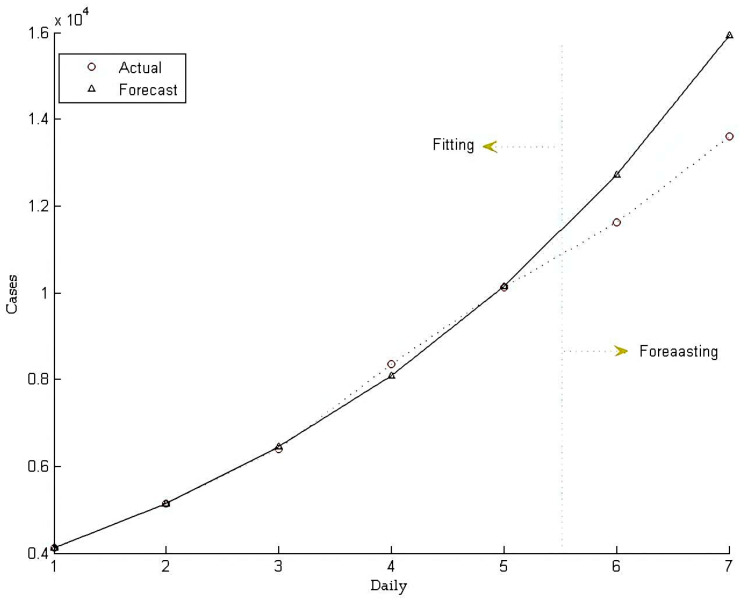
Forecasting effect of the GM (1, 1) model.

Table 5 shows that the related test parameters, including stepwise ratio, mean relative error, ratio of mean square error, small error probability, and relevancy degree, all meet the requirements, thereby confirming the excellent effect of the constructed forecasting model. These test data indicate that the GM (1, 1) model, namely Equation (13), can efficiently predict future data. The forecasted value series obtained by Equation (13) are presented in Table 6, and the forecasting effect is depicted in Figure 4.

As shown in Table 6, the number of new cases amounted to 2571 up to February 6 and 3225 on February 7, leaving a total of 5796. The permanent resident population in Wuhan as of 2020 was 12,326,600. Therefore, about 4.7 persons out of every 10,000 in Wuhan were infected with COVID-19 (ρ = 4.7). Combined with the results shown in Table 4, the total number of newly infected persons across different districts from February 6 to 7 is presented in Table 7 (all numbers are rounded-off, unit: persons).

The number of infections in Wuhan during the initial phase of the COVID-19 pandemic witnessed an explosive growth that subsequently resulted in a considerable shortage of emergency medical facilities. At the start of 2020, the construction of the Huoshenshan Hospital was commenced in Wuhan. This was followed on 26 January by the construction of Leishenshan Hospital, another large-scale emergency medical facility at Huangjiahu, Jiangxia District, which is an ideal place for quarantine due to its adjacency to a lake. Convention and exhibition centers and indoor stadiums were also temporarily repurposed as cabin hospitals to facilitate the prevention and control of COVID-19. Given the great importance of a timely emergency medical facility construction, the location-allocation decisions for newly built and rebuilt mobile cabin hospitals were made based on the forecasted demand for cabin hospitals and in consideration of the time, cost, and effect to rapidly receive and cure patients and to prevent the further spread of the virus. According to the location-allocation model in Section 3.3, t = 0.01 h per person per kilometer was set. Meanwhile, the parameters of GA were set as Po = 300, GP = 0.9, Maxgen = 500, and Pc = 0.7. Meanwhile, Pm was set to 0.07 in the early algorithm phase and 0.01 in the later algorithm phase. On the basis of the abovementioned GA, these tests were implemented in MATLAB on a PC with 1.83 GHz CPU and 1 GB RAM.

**Figure 5:**
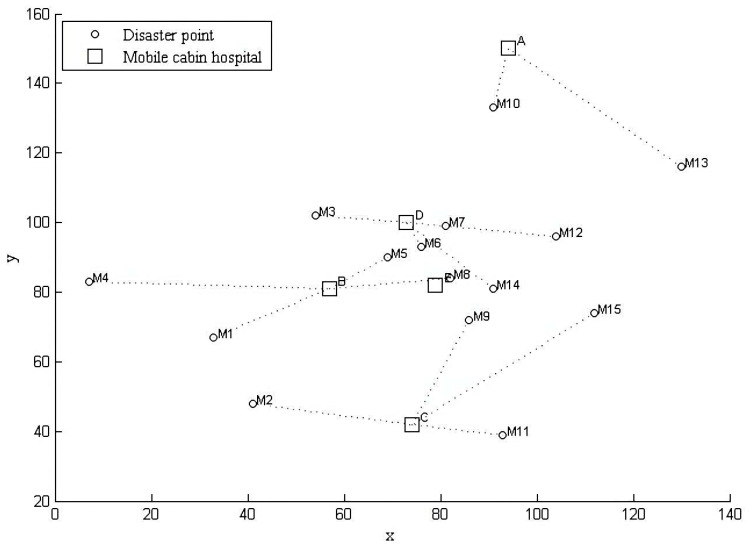
Cabin hospital location-allocation plan of SGA in Wuhan.

A cabin hospital location-allocation scheme was obtained using the traditional SGA, and the concrete optimized decision network is displayed in Figure 5.

**Figure 6:**
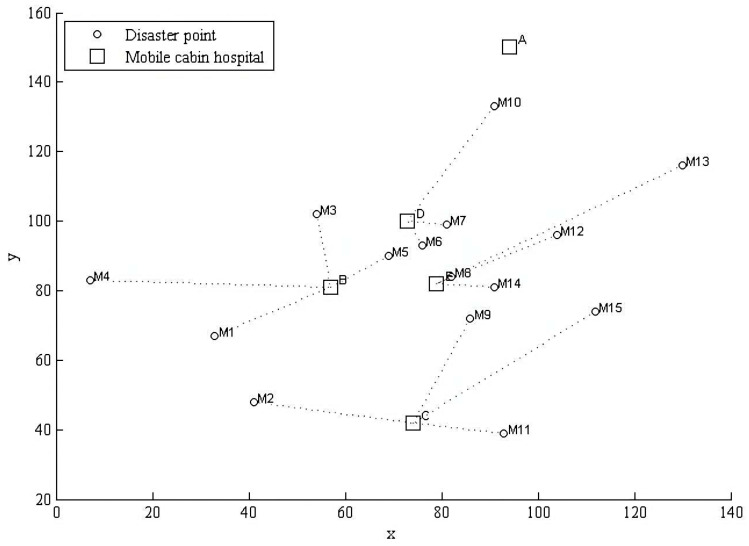
Cabin hospital location-allocation plan of SNCGA in Wuhan.

Another cabin hospital location-allocation scheme was acquired using SNCGA, and the specific optimized decision network is shown in Figure 6.

### 4.3. Comparison and Analysis of Calculation Results

To test its performance, more experiments are needed. First, SNCGA was compared with the classical SGA using the same parameters. The calculation results of these two algorithms are presented in Table 8. Second, the different random numbers with the maximum number of iterations are used to statistically test the two algorithms, and the test results are shown in Table 9. Third, the study increases the size of the problem by randomly adding one mobile cabin hospital each time, a total of five times. The *x*-axis coordinates of the five newly added mobile cabin hospitals are 30 km, 45 km, 100 km, 25 km, and 80 km, respectively; the *y*-axis coordinates of that are 120 km, 70 km, 90 km, 60 km, and 55 km, respectively. The construction time of these new mobile cabin hospitals is 145 h, and each has a maximum capacity of 1000 people. The statistical test results of the two algorithms are shown in Table 10.

Table 8 shows that under the same parameters, the calculation time of SNCGA was 20.25% shorter than that of SGA, whereas the total time spent by its cabin hospital location-allocation scheme was 8.34%. Table 9 shows that under the same parameters, the calculation time of SNCGA was shorter than that of SGA, whereas the total time spent by its cabin hospital location-allocation scheme was shorter as iteration is increased. The means of the calculation time for SNCGA was 19.10% shorter than that of SGA, whereas the means of the total time spent by its cabin hospital location-allocation scheme was 10.02% shorter, and the standard deviations of the calculation time for SNCGA were 29.21% shorter than that of SGA, whereas the standard deviations of the total time spent by its cabin hospital location-allocation scheme were 78.08% shorter. Table 10 shows that the calculation time of SNCGA was shorter than that of SGA, whereas the total time spent by its cabin hospital location-allocation scheme was shorter as the number of cabin hospital is increased. The means of the calculation time for SNCGA was 24.40% shorter than that of SGA, whereas the means of the total time spent by its cabin hospital location-allocation scheme was 5.02% shorter, and the standard deviations of the calculation time for SNCGA were 76% shorter than that of SGA, whereas the standard deviations of the total time spent by its cabin hospital location-allocation scheme were 213.24% more. The above results confirm that SNCGA has a better calculation effect than SGA. Given that the serial-number-coding scheme is convenient for genetic operation, using dynamic mutation is most appropriate in the solving process. The mutation rate was relatively high at the early stage of algorithm, and the searching solution space was expanded to ensure huge differences between the individual optimal value of every generation and the mean value of an entire generation. By contrast, the mutation rate was relatively low at the late stage of algorithm in order to gradually reduce the difference between the individual optimal value of each generation and the mean value of an entire generation, which would increase the rate of convergence. SNCGA can therefore avoid the early-maturing problem of SGA.

## 5. Conclusions

To relieve the medical system pressure in disaster-struck areas during major public health emergencies, reduce the risk of personnel cross-infection, and reduce the time consumed by emergency medical facility location-allocation systems, the emergency medical facility LAP was explored in this study, and the required number of emergency medical facilities was inferred based on the forecasting results obtained by a grey forecasting model, thus optimizing the emergency medical facility location-allocation scheme. The grey forecasting model was also analyzed, and the results obtained by the traditional GA for the emergency medical facility location-allocation scheme were compared with those obtained by the improved GA. The following conclusions were drawn:

1. The stepwise ratio test results obtained using the COVID-19 data in Wuhan, China, verify the excellent modeling effect of the grey forecasting model. Meanwhile, the mean relative error, ratio of mean square error, small error probability, and relevancy degree all indicate that the grey forecasting model has high accuracy and is suitable for calculating the number of emergency medical facilities demanded.
2. SGA has a longer computation time than SNCGA mainly because the former indicates whether emergency facilities are established by means of binary coding, which requires users to code the emergency facilities allocated to disaster points. In this case, the length of each chromosome string in SGA is longer than that in SNCGA, thereby consuming large memory, requiring a long computation time, and resulting in a low computation efficiency.
3. When using dynamic mutations, the SNCGA shows a better effect than SGA, which suggests that the convergence rate is reversely correlated with mutation rate. The main reason for this finding is that the serial number coded scheme adopted by the improved GA is convenient for genetic operation, thus making the dynamic mutation particularly suitable in the solving process. This improved GA not only improves the searching solution space ability of the algorithm but also has very strong stability and does not generate any adverse effect on the convergence characteristics. Therefore, SNCGA can avoid the early-maturing problem of the traditional SGA.
4. The emergency medical facility location-allocation scheme in SNCGA is generally better than that in SGA. Given that the characteristic advantages of the serial number coding rule are fully exerted by the improved GA, the problem can be naturally described by coded character sets, thus facilitating the handling of complex constraint conditions for decision variables and greatly simplifying the computation of the problem fitness function. When used, the SNCGA can optimize the emergency medical facility location-allocation scheme to a greater extent within the minimum possible time.

This study forecasted the number of recently accumulated COVID-19 cases during the onset of the pandemic by using an appropriate forecasting method. On the basis of the obtained forecasts, the number of emergency medical facilities demanded was calculated, and a corresponding emergency medical facility (i.e., mobile cabin hospitals) LAP optimization model was constructed as a mathematical optimization model and solution to the LAP of emergency medical facilities during the COVID-19 pandemic. However, the first issue, given the complex factors influencing the spread of the virus, is that future research should construct a model that forecasts the turning point of a pandemic and establish a corresponding dynamic multi-stage cabin hospital location-allocation optimization model according to the dynamic evolution trends of this pandemic, and the second one is that a standard solver can be used to solve the problem to verify its correctness in the future study.

## Figures and Tables

**Table 1:** Related parameters of standby mobile cabin hospitals.

S/N	A (Newly Built)	B (Rebuilt)	C (Newly Built)	D (Rebuilt)	D (Rebuilt)
Coordinates (*x*, *y*)	(94, 150)	(57, 81)	(74, 42)	(73, 100)	(79, 82)
Construction time (h)	288	72	288	72	48
Maximum capacity	2000	1500	2000	1500	1200

**Table 2:** Coordinate parameters of disaster points in Wuhan, China.

S/N		M1	M2	M3	M4	M5	M6	M7	M8	M9	M10	M11	M12	M13	M14	M15
Coordinates	*x*	33	41	54	72	69	76	81	82	86	91	93	104	130	91	112
	*y*	67	48	102	83	90	93	99	84	72	133	39	96	116	81	74

**Table 3:** Accumulative number of infected cases in Wuhan, China.

$X^{\left(0\right)}$	February 1	February 2	February 3	February 4	February 5
Accumulative cases (persons/day)	4109	5142	6384	8351	10,117

**Table 4:** Permanent resident populations in different districts of Wuhan, China.

$\vartheta_{m}$	M1	M2	M3	M4	M5	M6	M7	M8	M9	M10	M11	M12	M13	M14	M15
Population	55	48	85	84	67	65	97	109	173	115	97	46	86	12	94

**Table 5:** Related model test parameters.

Index	minL(k)	maxL(k)	$\bar{\Delta }$	μ	φ	$\gamma_{s\beta }$
Calculation results	0.7645	0.8254	0.009	0.054	1	0.993
Criteria	≥0.7165	≤1.3956	≤0.01	≤0.35	≥0.95	≥0.90

**Table 6:** Accumulative number of infected cases in Wuhan forecasted using the GM (1, 1) model.

${\hat{x}}^{\left(0\right)}(k)$	February 1	February 2	February 3	February 4	February 5	February 6	February 7
Accumulative cases (persons/day)	4190	5146	6451	8087	10,137	12,708	15,930

**Table 7:** Total number of new cases in Wuhan, China, on February 6 and 7.

$q_{m}$	M1	M2	M3	M4	M5	M6	M7	M8	M9	M10	M11	M12	M13	M14	M15
Infected cases	259	226	400	395	315	306	456	512	813	541	456	216	404	56	442

**Table 8:** Calculation results of SNCGA and SGA.

Item	Standard GA	Serial Number Coded GA	Percentage
Emergency facility location-allocation scheme	Cabin hospital A: M10, M13	Cabin hospital A: None	
	Cabin hospital B: M1, M4, M5, M8	Cabin hospital B: M1, M3, M4, M5	
	Cabin hospital C: M2, M9, M11, M15	Cabin hospital C: M2, M9, M11, M15	
	Cabin hospital D: M3, M6, M7, M12, M14	Cabin hospital D: M6, M7, M10	
	Cabin hospital E: None	Cabin hospital E: M8, M12, M13, M14	
Total time	2322.36 h	2128.73 h	−8.34%
Computation time	93.22 s	74.34 s	−20.25%

**Table 9:** Calculation results of SNCGA and SGA for different random numbers of iterations.

Maxgen	Item	Standard GA	Serial Number Coded GA	Percentage
100	Total time	2571.60 h	2207.26 h	−14.17%
	Computation time	20.62 s	19.50 s	−5.43%
200	Total time	2549.53 h	2184.28 h	−14.33%
	Computation time	39.24 s	34.10 s	−13.1%
300	Total time	2401.46 h	2179.68 h	−9.24%
	Computation time	56.06 s	47.26 s	−15.7%
400	Total time	2351.63 h	2168.46 h	−7.79%
	Computation time	78.48 s	67.07 s	−14.54%
500	Total time	2322.36 h	2128.73 h	−8.34%
	Computation time	93.22 s	74.34 s	−20.25%
600	Total time	2278.79 h	2157.19 h	−5.34%
	Computation time	126.96 s	93.11 s	−26.66%
	Means	2412.56 h	2170.93 h	−10.02%
		69.10 s	55.90 s	−19.10%
	Standard deviations	121.59	26.65	−78.08%
		38.55	27.29	−29.21%

**Table 10:** Calculation results of SNCGA and SGA for the increase of the problem size.

The Number of Cabin Hospital	Item	Standard GA	Serial Number Coded GA	Percentage
6	Total time	2221.82 h	2184.28 h	−1.69%
	Computation time	98.14 s	80.79 s	−17.68%
7	Total time	2204.96 h	2179.68 h	−1.15%
	Computation time	102.75 s	83.94 s	−18.31%
8	Total time	2184.28 h	2049.99 h	−6.15%
	Computation time	110.34 s	84.52 s	−23.40%
9	Total time	2183.20 h	1989.69 h	−8.86%
	Computation time	121.24 s	87.43 s	−27.89%
10	Total time	2136.73 h	1978.84 h	−7.39%
	Computation time	129.99 s	88.79 s	−31.69%
	Means	2186.20 h	2076.50 h	−5.02%
		112.55 s	85.09 s	−24.40%
	Standard deviations	31.94	100.05	213.24%
		13.04	3.13	−76%

## Data Availability

Not applicable.
